# Gallic Acid Induces HeLa Cell Lines Apoptosis via the P53/Bax Signaling Pathway

**DOI:** 10.3390/biomedicines12112632

**Published:** 2024-11-18

**Authors:** Umut Sarı, Fuat Zaman, İlhan Özdemir, Şamil Öztürk, Mehmet Cudi Tuncer

**Affiliations:** 1 Department of Gynecology and Obstetrics, Umut Sarı Clinic, 34371 Istanbul, Turkey; sarifuat77@gmail.com; 2 Department of Obstetrics and Gynecology, Diyarlife Hospital, 21100 Diyarbakır, Turkey; fuatzaman@hotmail.com; 3 Private Buhara Hospital, Gynecology and Obstetrics Clinic IVF Center, 25070 Erzurum, Turkey; ilhanozdemir25@yandex.com; 4 Vocational School of Health Care Services, Çanakkale Onsekiz Mart University, 17100 Çanakkale, Turkey; ozturksamil@outlook.com; 5 Department of Anatomy, Faculty of Medicine, Dicle University, 21200 Diyarbakir, Turkey

**Keywords:** cervical cancer, cytotoxicty, doxorubicin, anticancer, natural products

## Abstract

Background: Cervical cancer is a type of cancer that originates from the endometrium and is more common in developed countries and its incidence is increasing day by day in developing countries. The most commonly prescribed chemotherapeutic drugs limit their use due to serious side effects and the development of drug resistance. For this reason, interest in new active ingredients obtained from natural products is increasing. This study aimed to reveal the apoptotic and antiproliferative effects of gallic acid and doxorubicin combination therapy against the HeLa cell line. Methods: We investigated the anti-cancer effects of doxorubicin and gallic acid in the human HeLa cervical cell line by using the MTT test, Nucblue staining for the identification of apoptotic cells due to nuclear condensation using fluorescent substance, and apoptotic markers P53 and Bax for the RT-PCR test. Results: The highest cytotoxic effect obtained in the study, the highest increase in apoptotic induction, and a significant difference in P53/Bax levels were seen in the gallic acid/doxorubicin combination. Additionally, it was determined that gallic acid exhibited an effective cytotoxic effect on HeLa and HaCat cells within 48 and 72 h of application. Conclusions: The obtained findings show that the gallic acid/doxorubicin combination applied to HeLa cells may be an alternative treatment against both the cytotoxic effect size and the side effects of the chemotherapy agent.

## 1. Introduction

Cervical cancer remains a significant global health burden, with an estimated 570,000 new cases and 311,000 deaths reported annually worldwide. Despite advancements in screening and treatment modalities, the mortality rate associated with cervical cancer remains high, underscoring the urgent need for novel therapeutic interventions. Apoptosis, or programmed cell death, plays a crucial role in maintaining tissue homeostasis by eliminating damaged or aberrant cells. Dysregulation of apoptosis is a hallmark of cancer, including cervical carcinoma, where evasion of cell death contributes to tumor progression and therapeutic resistance.

The HeLa cell line is the first immortalized human cell line derived from the tumor of a cervical cancer patient named Henrietta Lacks. Although she died of cancer in 1951, the HeLa cell line has been consistently augmented in culture and has become one of the most widely used human cell lines in biomedical research [1]. Cervical cancer is one of the cancers that threaten the lives of approximately 500,000 women annually. Risk factors include smoking, human papillomavirus, and immune system disorders. Although treatment can be easily applied in the early stages, long-term morbidity resulting from treatment is also common [2].

Although the war against cancer worldwide has been fought for years, its morbidity and mortality have not decreased. Every year, enormous amounts of money are spent on cancer research and various methods are developed for its treatment, but a decrease in the incidence of the disease cannot be achieved. The main reason for this is that most of the drugs used in the treatment of cancer often cause negative side effects that further hinder the treatment processes in vital organs such as the heart and liver and eventually lead to the progression of cancer [3]. In addition, the resistance of cancer cells to radiotherapy and chemotherapy is not a desired condition for the success of the treatment. Therefore, it is important to discover new treatment strategies or chemotherapeutics with minimal side effects, easy accessibility, lower cost, and higher efficiency for the management of this deadly disease [4].

Doxorubicin (DOX), an anthracycline derivative antibiotic, is obtained from Streptomyces peucetius [5,6] in its tetracyclic aglycone structure to which an amino sugar is attached. It is one of the important antitumor drugs with clinical application in the treatment of breast, ovary, testicular, thyroid, lung cancers, and many sarcomas. Doxorubicin disrupts DNA replication and RNA transcription by intercalating into double-stranded DNA and also causes DNA damage by binding to the topoisomerase II enzyme. Doxorubicin, a powerful antineoplastic drug used in the treatment of many types of cancer, has side effects such as hematopoietic suppression, nausea, vomiting, and alopecia that prevent its clinical use. The most important side effect requiring dose limitation is cardiotoxicity and may occur in bone marrow suppression [7,8].

Gallic acid (GA) is a polyhydroxyphenolic compound naturally found in apples, grapes, strawberries, and many other fruits, which are among our daily dietary habits [9]. Recent studies have shown that GA and its derivatives have both in vivo and in vitro anticancer potential [10]. Previous studies on the anti-cancer properties of GA have reported positive results in many cancer cells such as human ovarian cancer [11,12,13]. It has been reported that the anticancer effect of this polyphenol compound is due to its ability to stop cell proliferation and kill cancer cells by inducing apoptosis [14,15].

In this study, we investigate the potential of GA to inhibit apoptosis in HeLa cervical cancer cell lines through modulation of the P53/Bax signaling pathway. Through a comprehensive series of experiments, including cell viability assays, molecular analyses, and functional assays, we aim to elucidate the molecular mechanisms underlying the anti-apoptotic effects of GA. Our findings have the potential to not only enhance our understanding of the therapeutic potential of GA but also to provide valuable insights into the development of novel therapeutic approaches for the management of cervical cancer and other malignancies characterized by dysregulated apoptosis. With the HeLa cell line, the possible synergistic or antagonistic effects of DOX and GA on molecules that play an important role in the apoptotic signaling pathways, P53/Bax expression, and cell proliferation/migration were investigated.

## 2. Material and Methods

### 2.1. Cell Culture

Nine different concentrations were used, ranging from 10 to 1000 µM GA and 10–1000 nM DOX applied to HeLa and HaCat cell lines. The HeLa cell line was obtained from the American Type Culture Collection (ATCC^®^HTB-161^TM^, Manassas, VA, USA). Our other cell line is the HaCat cell line, which was obtained from Germany (CLS 300493, DKFZ, Heidelberg, Germany). The HeLa cell series were cultured in Eagle’s minimum essential medium (EMEM) (Sigma Aldrich, Darmstadt, Germany) and the HaCaT cell line was cultured in Dulbecco’s modified eagle medium (DMEM) (Invitrogen, Carlsbad, CA, USA) containing glutamine and penicillin-streptomycin solution, fetal bovine serum (FBS) (Gibco, Grand Island, NY, USA), and the cells were grown in sterile incubators at 37 °C and 5% CO_2_. Commercially available GA (Sigma) and DOX (Koçak Pharma, Istanbul, Turkey) were diluted with appropriate solvents to prepare 9 different concentrations and applied to HeLa and HaCat cells. The stock solutions of DOX and GA agents were prepared using ultrapure ethanol (Merck, Rahway, NJ, USA). Stock solutions of 5 mM for DOX and 100 mM for GA were made, portioned, and stored at −20 °C. In the applications, the final concentration of the vehicle in the flasks or plate wells was reduced to 1%.

### 2.2. MTT Assay

EMEM medium was used to feed and propagate HeLa cells, and DMEM medium was used to feed and propagate HaCat cells. Cells were grown and incubated in a 75 cm^2^ cell culture flask (Thermo Fisher Scientific, Waltham, MA, USA). The cell medium was changed every 3–4 days and cells were passaged when they reached 80–90% density. HeLa and HaCat cells were treated with concentrations of 10–1000 nM DOX and 10–1000 µM GA. The anticancer effect of GA and DOX was determined by the 3-(4,5-dimethylthiazol-2-yl)-diphenyltetrazolium bromide (MTT) test. In this context, HeLa and HaCat cells, which reached 80–90% density, were removed with Trypsin-EDTA, counted under an inverted microscope, and inoculated with multipipettes in 96-well culture dishes with an average of 3 × 10^3^ seeding cell density per well. The seeded cells were incubated for 24 h in a 5% CO_2_ incubator at 37 °C. After incubation, the cell medium was changed and different concentrations of DOX and GA were added to the wells containing the cells and incubated for 24, 48, and 72 h. After the incubation, the medium in the wells was removed and the MTT solution (20 μL) prepared in sterile PBS was added to each well; the microplates were incubated at 37 °C for 4 h. Afterward, the liquid part in the wells was withdrawn and the incubation was stopped by adding 200 µL of dimethyl sulfoxide (DMSO) (Merck) to each well. Optical densities of cells in microplates were determined by a spectrophotometer (Multiskan GO microplate reader (Thermo Scientific)) at wavelengths of 570 nm. The average absorbance value obtained from the measurement of the control wells was considered to represent 100% viable cells. Percentage (%) viability values were determined by comparing the absorbance values obtained from the microplate wells of DOX and GA, to which diluted concentrations (9 different concentrations in the range of 10–1000) were applied to the absorbance value obtained from the control group.

### 2.3. Determination of an Inhibitory Dose (IC_50_)

According to the cell viability results from the MTT analysis, the IC_50_ values of the vehicle group and the tested groups were calculated using a statistical program. The determined dose was used to analyze the results in more detail. Once the IC_50_ was determined, this determined dose was applied to analyze more specific results. After, the MTT procedure was applied. The plates were read spectrophotometrically at 570 nm wavelengths with a Multiskan GO microplate reader (Thermo Scientific). The value obtained from the vehicle-applied control group was determined as a comparative viability ratio based on 100% viability. In the control and experimental groups, each IC_50_ value for tumor cell lines and chemotherapy agents was calculated using probit analysis with the SPSS 20 statistical package program.

### 2.4. Apoptotic Assay

A Hoechst NucBlue^®^ Live ReadyProbes^®^ Reagent (Thermo Scientific) specific dye was used to determine nuclear morphology changes and apoptotic structures after apoptosis caused by DOX and GA agents in the HeLa cell line. In this context, HeLa cell lines were seeded in 24-well plates at 5 × 10^4^ cells/well, and the cells were incubated at 37 °C under 5% CO_2_ conditions. The following day, vehicle, doxorubicin, gallic acid, and doxorubicin + gallic acid were added to these wells using the IC_50_ values obtained from 48-h incubation in the MTT assay. The Hoechst staining solution was prepared by diluting the Hoechst stock solution 1:2000 in PBS. The staining was performed directly as live cell staining in accordance with the kit protocol and the cells were incubated at 37 °C for 30 min. After this period, the plates were photographed using a DAPI filter at ×20 objective magnification using the Thermo EVOS^®^ FL Imaging System using the brightfield mode and fluorescent mode. In addition, unstained cells were imaged and examined by phase contrast microscopy.

### 2.5. Determination of Gene Expression by RT-PCR

Quantitative gene analysis was performed to observe the effect of GA applied to HeLa cells on anti-apoptotic and pro-apoptotic gene expression levels. For this purpose, primer sets specific for target sequences were designed. The β-actin gene was taken as a reference to evaluate the relative expression change. The level of change in expression was calculated by comparison between threshold cycle (CT) values.

### 2.6. Total RNA Isolation

For total RNA isolation, 1 mL of cold PBS was added to each well of 6-well microplates containing HeLa cells treated with the IC_50_ value of DOX, GA, and DOX+GA and then cells were scraped from the wells. RNA was isolated from the samples 72 h after the application. The Purelink RNA mini kit (Thermo) was used in the isolation phase. Accordingly, 1% mercaptoethanol was added to the lysis solution included in the kit, the medium was removed, and 1 mL of this solution was placed in each 25-mL flask washed with D-PBS. These flasks were kept in a 37 °C incubator for 20 min. Afterward, the cells were collected in 2 mL Ependorf tubes and an equal volume of 1 mL of 70% ultrapure ethanol (Merk) was added to them. It was centrifuged sequentially at 12,000× *g* for 30 s. RNAs were loaded onto the column and centrifuged first with washing solution 1, then twice with washing solution 2, at 12,000× *g* for 30 s at each stage. After the washing procedures, the columns were centrifuged once at 12,000× *g* for another 3 min to dry them. Then, the columns were placed in sterile new 1.5 mL Ependorf tubes, and 60 µL of the solution given in the kit was pipetted to the middle of the membrane in the column, and these columns were centrifuged at 12,000× *g*. Pure RNAs were collected in an Ependorf tube by centrifugation for 1 min. The purity of the collected RNAs was determined by an Optizen NanoQ microvolume spectrophotometer (Mecasys, Daejeon, Republic of Korea) and all were equalized with ultrapure water to 750 ng/10 µL. Total RNA isolation from HeLa cells was performed according to the manufacturer’s protocol (Thermo Fisher Scientific). cDNA samples were stored at −20 °C and mRNA samples were stored at −86 °C.

### 2.7. cDNA Synthesis

Complementary DNA synthesis was performed to enable the RNAs obtained after the synchronization process to be amplified by PCR. At this stage, a High-Capacity cDNA Reverse Transcription Kit (Life Technologies, Carlsbad, CA, USA) was used. According to the kit protocol, the enzyme, DNTP mix, and random primers in the kit were mixed and pipetted into PCR tubes as 10 µL. Afterward, the total RNA, which was equal to 750 ng/10 µL in the previous section, was put into the same tubes. These tubes were incubated in the Applied Biosystems^®^ ProFlex™ PCR System thermal cycler step 1 at 25 °C, 10 min; step 2 37 °C, 120 min; step 3 cDNA synthesis was performed using 85 °C, 5 min cycles. The cDNAs obtained were stored at −20 °C for ongoing studies.

### 2.8. Quantitative Real-Time PCR Study (qRT-PCR)

cDNAs were amplified using the qRT-PCR technique and SYBR Green (2xqPCRBIO SyGreen Mix Lo-ROX Kit, PCR Biosystems, London, UK). In the study, the expression levels of BAX and P53 genes responsible for the apoptosis pathway were analyzed by the qRT-PCR method in the control and tested groups of HeLa cervical adenocarcinoma cells. β-Actin was taken as a reference as a housekeeping gene in qPCR studies. The expression of selected genes and housekeeping genes were analyzed in a Q-PCR device (Bio-Rad CFX96). The groups that did not receive any treatment were used as controls. qPCR reaction conditions: incubation at 95 °C for 2 min; 5 s at 95 °C, 40 cycles; 45 s at 66 °C, 45 cycles. After this, it was set at 74 °C for 2 min, 45 cycles, and finally at 72 °C for 5 min, 1 cycle. mRNA expression levels were determined by the 2^-ΔΔCt^ method [16]. In the experiments, all cDNA samples and standard samples were analyzed in triplicate in the same group and under the same conditions to reduce experimental errors and differences. Endogenous control GAPDH (glyceraldehyde 3-phosphate dehydrogenase) and β-actin mRNA expressions were used as calibration and correction factors with multiple control methods. The following primers for changes in the expression of these genes were provided from the 5′ end to the 3′ end (Table 1).

### 2.9. Protein–Protein Interaction (PPI) Analysis

PPI data were retrieved from the STRING database. The STRING database provides descriptions of protein–protein interactions (PPIs) as well as confidence intervals for data scores. A confidence score greater than or equal to 0.4 was chosen to construct the interaction network of proteins with target genes.

### 2.10. Enrichment Analysis

Data on the functional annotation of genes and the canonical pathways associated with the strong connections established with these proteins were obtained using the ShinyGO 0.80 program.

### 2.11. GO Functional Enrichment Analysis

Three types of Gene Ontologies (GO) were performed on possible target genes: cellular component (CC), biological process (BP), and molecular function (MF). The SRplot bioinformatics program was used to evaluate these data.

### 2.12. Statistical Analysis

After cell viability rates were determined with the MTT method, the averages of the expression values obtained by qRT-PCR were determined by one-way ANOVA, and a significant difference was detected with the Tukey HSD test. In comparisons between the two groups, the independent sample T-test was used, taking into account the homogeneity of the data. The results were found to be significant at *p* ≤ 0.05.

## 3. Results

### 3.1. Cell Viability

**Figure 1:**
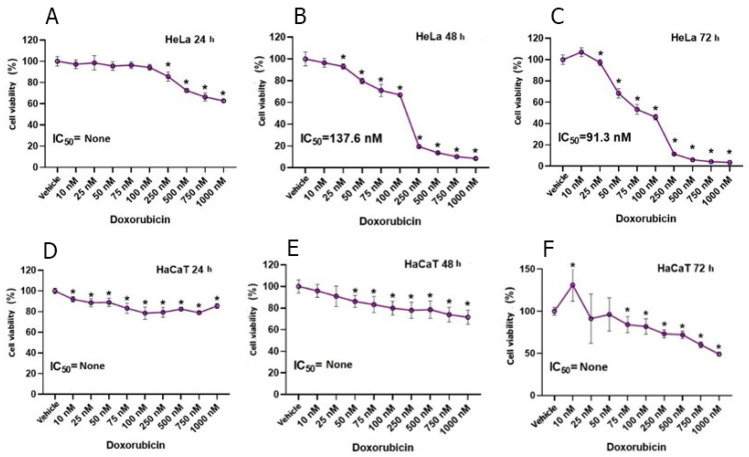
The effect of DOX application on 9 different concentrations obtained by serial dilution in the concentration range of 10-1000 nM in HeLa cervix adenocarcinoma (**A**–**C**) and HaCaT human skin keratinocyte cell line (**D**–**F**) on compared to the vehicle group and the IC50 value of the chemotherapy agent (n = 6; data are mean ± standard deviation values, inhibition concentration (IC) values calculated by probit analysis). * Data are statistically significant compared to control, one-way ANOVA, Tukey HSD test, *p* ≤ 0.05.

**Figure 2:**
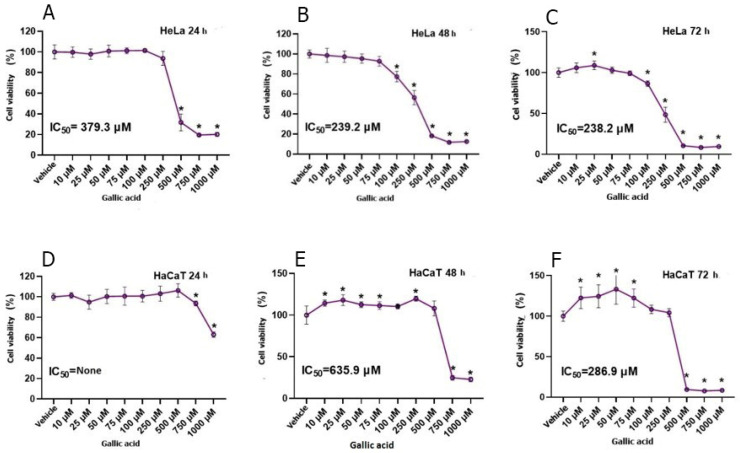
Effect of GA application on 9 different concentrations obtained by serial dilution between 10-1000 µM concentration range in HeLa cervix adenocarcinoma (**A**–**C**) and HaCaT human skin keratinocyte cell line (**D**–**F**) cell lines for 24, 48 and 72 hours on cell viability compared to the vehicle group and the IC50 value of GA (n = 6; data are mean ± standard deviation values, inhibition concentration (IC) values calculated by probit analysis). * Data are statistically significant compared to control, one-way ANOVA, Tukey HSD test, *p* ≤ 0.05.

In the study, DOX and GA were applied to HeLa and HaCaT cell lines at nine different concentrations for 24, 48, and 72 h. The data obtained within the scope of the study could not find the IC_50_ value in 24 h of DOX application to the HeLa cell line. It was observed that there was a significant decrease in cell proliferation inversely to the increase in DOX dose. At initial values with a cell density of 100,000, the average number of cells was determined to be 65,000 until the application of DOX at a concentration of 1000 nM. After 48 h of DOX application to HeLa cells, the IC_50_ value was determined as 137.6 nM. The average cell viability in 48 h of DOX application was determined as 55.93. In 72-h DOX application to HeLa cells, IC_50_ was determined as 91.3 nM, while the average cell viability was determined as 49.72 (Figure 1). As a result of statistical analysis, it was determined that the 72-h IC_50_ value occurred after 25 nM DOX application. IC_50_ could not be determined in 24, 48, and 72-h DOX application to HaCaT cells. The IC_50_ value in GA acid-treated HeLa cells exhibited a decrease with increasing exposure time (Figure 2). The average 24-h HeLa cells’ cell viability was determined as 76.64, the 48-h cell viability was determined as 66.03, and the 72-h cell viability was determined as 67.99. In GA application to HaCaT cells, IC_50_ values could not be determined for 24, 48, and 72 h (Figure 2). However, a decrease in cell viability was observed parallel to the increase in GA dose. The viability rate in HaCaT cells was observed as 96.35 in the 24-h application, 94.20 in the 48-h application, and 84.03 in the 72-h application. After the statistical determination of the IC_50_ value, the viability of the HeLa cell line decreased significantly. When the GA application and DOX application were compared, it was shown that the viability in the HeLa cell line decreased more in the DOX group.

### 3.2. NucBlue Staining Results

**Figure 3:**
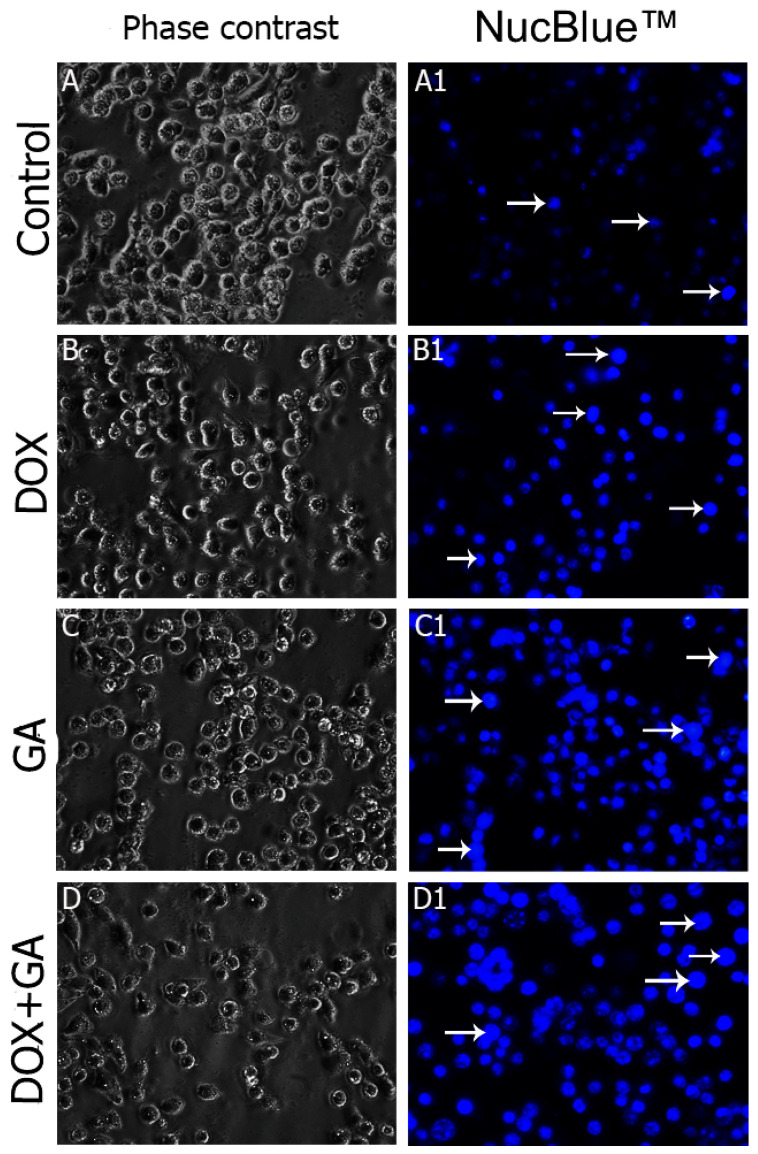
Cell morphology, nuclear structure, and apoptotic body formation (magnification: ×20) in HeLa cervical adenocarcinoma cell populations treated for 48 hours with vehicle control (**A**,**A1**), DOX IC50: 137.6 nM (**B**,**B1**), GA IC50: 239.2 μM (**C**,**C1**), and DOX IC50+GA IC50 (**D**,**D1**) (Arrow: apoptotic cell).

**Figure 4:**
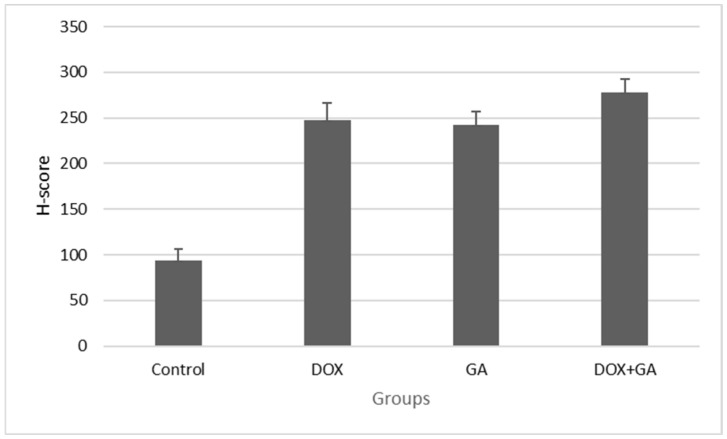
H-scores were derived from semi-quantitative assessments of both staining intensity (scale 0–3) and the percentage of positive cells (0–100%) and, when multiplied, generated a score ranging from 0 to 300.

NucBlue staining was performed on GA IC50-, DOX IC50-, and DOX+GA IC50-applied samples in order to confirm the results of the MTT analysis and to determine whether the detected cell deaths were apoptotic. The apoptotic staining results showed that cell death increased in samples to which GA IC50, DOX IC50, and DOX+GA IC50 were applied. When semiquantitative microscopic findings were compared with MTT analysis results, it was understood that apoptosis was the cause of cell death. In NucBlue staining, GA IC50 and DOX IC50 doses were determined to suppress cell proliferation and thus cause a decrease in cell number. An increase in nuclear fragmentation is an indication that apoptosis has occurred. Cell images in the light microscope also support these findings (Figure 3). When the cells showing positivity with Nucblue staining were evaluated semi-quantitatively, a statistically significant difference was detected between the control group and the DOX IC50- and GA IC50-applied groups (Figure 4).

### 3.3. Quantitative Real-Time PCR Analysis Results of Apoptotic Markers

In the continuation of the study, DOX and GA IC_50_ were applied for 48 h in the experimental groups determined by MTT analyses and NucBlue staining in Quantitative Real-Time PCR analyses. RT-qPCR experiments were performed on a total of 9 samples in all experimental groups; Proapoptotic P53 and Bax gene expressions were normalized to β-actin expression of the same sample, which was used as the internal control gene. P53, Bax, and β-actin gene expressions were determined at detectable levels, and amplification curves were created. The amplification curves of these genes were determined by the number of cycles on the x-axis and the Rn value on the y-axis.

**Figure 5:**
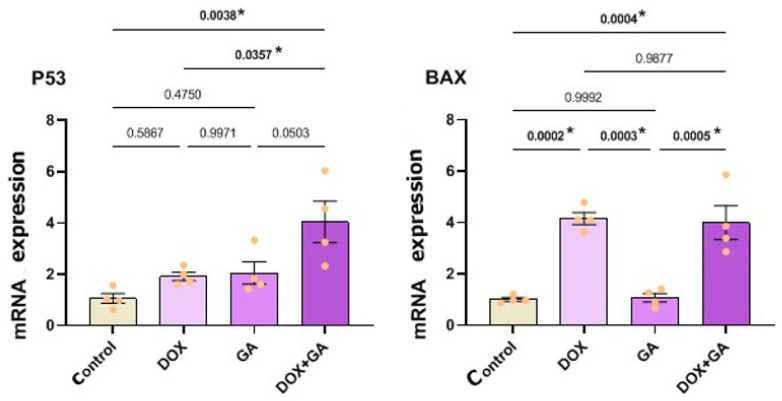
Relative fold increases values of P53 and BAX gene expressions in HeLa cervical adenocarcinoma cell lines, DOX IC_50_: 137.6 nM, GA IC_50_: 239.2 μM, 48 h, after single and combined drug administration (data in multiple control with β-actin and GAPDH mRNA level). Method, n = 4 data mean ± SH), * means are statistically different, one-way ANOVA, Tukey HSD test, *p* values are given in the graph.

As a result of the calculations, P53 and Bax increased very little in the 48 h control group. Although P53 gene expression was determined at a detectable level in the group in which only DOX was administered for 48 h, it was not statistically significant in the control group. A statistically significant difference was found between the control and DOX+GA and DOX and DOX+GA groups in P53 gene expression. In the DOX group, it was determined that Bax gene expression (RQ = 0.5) was expressed at the highest level with an increase of 50%. Bax gene expression in the DOX-treated group showed a statistically significant difference compared to the control and GA-treated groups. The significance of *p* ˂ 0.0002 between DOX and the control group and *p* ˂ 0.0003 between DOX and GA was determined. No statistical difference was found between DOX and DOX+GA groups. When compared with other groups, no significance could be found in P53 gene expression in only the GA-applied group. However, a statistically significant difference was found between the control group and the DOX+GA group in Bax gene expression (Figure 5). It was determined that P53 and Bax protein levels increased significantly with GA and DOX treatment. Especially in the GA+DOX group, the P53 level increased to the maximum, while Bax increased the most in the DOX group (Figure 5).

### 3.4. Protein–Protein Interaction (PPI) (Bax, P53)

**Figure 6:**
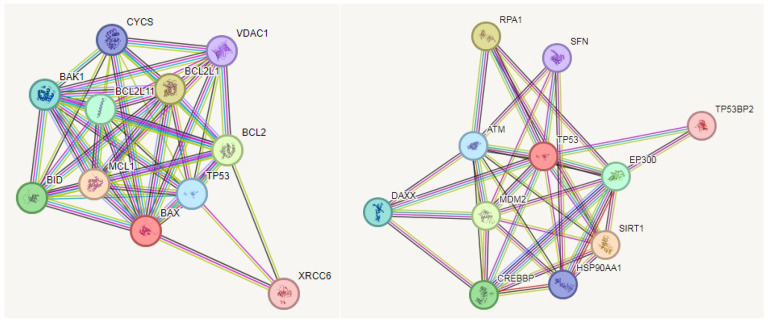
PPI and interaction between various genes of cervical cancer.

Predictions from STRING analysis were used to depict protein interactions. The visualization showed 11 nodes, 47 edges, an average node degree of 8.55 for the BAX gene, 11 nodes, 35 edges, and an average node degree of 6.36 for the P53 gene (Figure 6). Based on nodal degree, the following genes were identified as the top 10 central genes for BAX and P53: CYCS, VDAC1, BAK1, BID, MCL1, XRCC6, BCL2, BCL2L11, BCL2L1, TP53 and RPA1, ATM, DAXX, CREBBP, HSP90AA1, EP300, TP53BP2, SFN, MDM2, and SIRT1. These targets are hypothesized to be the primary targets in cervical cancer of GA.

### 3.5. KEGG Pathway

**Figure 7:**
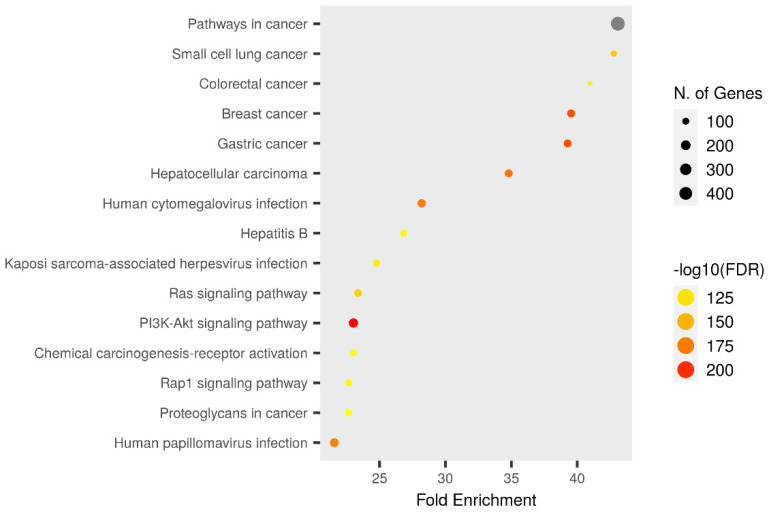
Enrichment analysis for the 530 common compound targets in cancer pathway.

**Figure 8:**
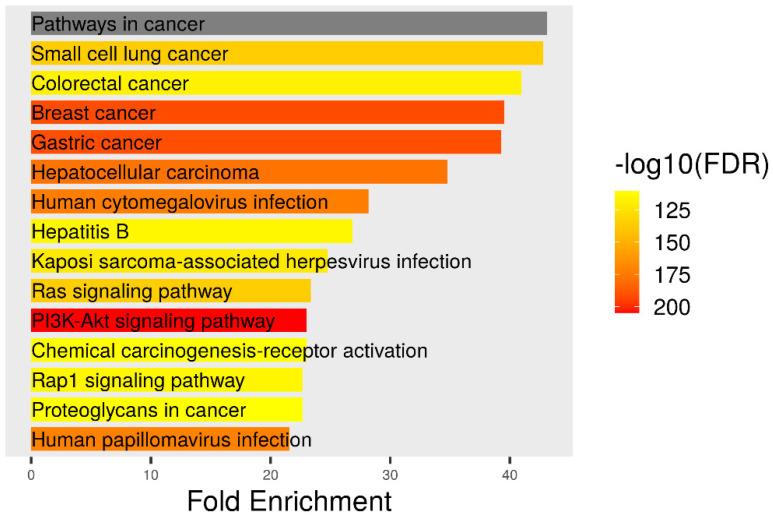
Enrichment analysis for the 331 common compound targets in human papilloma virüs infection and cervical cancer.

KEGG pathway enrichment analysis of target genes was performed with the Shiny 0.80 program. The findings showed that 530 genes were involved in the enrichment process and 331 pathways were human papillomavirus infection and cervical cancer-related, exhibiting a significant correlation with target genes (*p* < 0.05) (Figure 7 and Figure 8).

## 4. Discussion

In the present study, we investigated the molecular mechanisms by which GA exerts its effects on HeLa cells, specifically focusing on the P53/Bax signaling pathway. Our findings reveal that GA significantly influences the regulation of apoptosis in these cells, acting through the modulation of key apoptotic markers. This pathway, well-known for its critical role in cellular stress responses and apoptosis, appears to be a major target of GA, suggesting its potential as a therapeutic agent in cervical cancer treatment. The apoptotic and cytotoxic effects of both DOX, which is frequently used in malignancies, and GA, whose anticancer effects have been detected in many types of cancer in recent years, on HaCaT and HeLa cell lines in vitro and the potential therapeutic consequences of targeting the P53/Bax axis in the treatment of cervical cancer were discussed [13,14]. In addition, we contextualize our findings within the broader scope of GA research and suggest avenues for future research aimed at exploiting its therapeutic potential in combating cervical cancer and other malignancies characterized by dysregulated apoptosis.

Today, many studies and treatment methods are being developed for cancer treatment, and among these methods, the most effective treatment is still provided by chemotherapeutic drugs and radiotherapy. However, although these treatments have a very important place, their application is limited due to the potential to damage vital organs such as the heart and liver and cause side effects such as drug resistance [5]. For this reason, every natural herbal product has become the focus of attention in cancer treatment and is seen as a way to overcome these problems. In this study, the cytotoxicity and antiproliferative effects of GA, which has a high anticancer, anti-inflammatory, and antioxidant potential [14,15,16] on the human HeLa cell line, were compared and combined with a powerful chemotherapeutic agent such as DOX, and its ability to be an alternative treatment was investigated and to potentiate the anti-cancer effects of GA/DOX combination was investigated by performing MTT method, apoptotic staining, and RT-PCR for proapoptotic P53 and Bax genes. Our results revealed that GA could be a promising treatment, associated with an MTT cytotoxic effect, the highest induction of apoptosis by apoptotic staining, and a significant increase in P53 and Bax gene expressions.

Plants containing natural compounds, especially flavonoids, are considered important in many cancer treatments as anticancer agents due to their strong therapeutic properties and limited toxicity to healthy cells. Many studies emphasize that flavonoids can be an important source of cancer treatment. It has been reported that they exhibit anticancer properties by controlling important mechanisms of cancer, such as cell proliferation, angiogenesis, and metastasis and by activating the apoptosis mechanism [16,17]. Apoptosis has been assumed to be a form of programmed cell death. In our studies, we also demonstrated the expression of proapoptotic genes P53 and Bax, which are involved in the apoptosis process induced by GA in HeLa cells. In studies investigating the cytotoxic effects of GA in doses ranging from 10 to 400 µM in various cell lines, the IC_50_ value was determined on Calu-6, A 549 (adenocarcinomic human alveolar basal epithelial cells), HeLa cells [18,19,20]. The results from these studies show that GA induces cell death in tumor cells. The findings obtained from the study showed that the proliferation activity of HeLa cells was reduced by GA and DOX treatment. In particular, the cytotoxic effect of GA on HeLa cells may be explained by its apoptosis-inducing properties when combined with potent chemotherapy agents. The reason why the time-dependent IC_50_ values of GA detected in this study differ from the IC_50_ values obtained in other cancer studies [21] is due to the fact that the MTT test, which has been used in cancer research for over 30 years, is rarely consistent for a given chemical. He et al. attributed the source of this problem to manufacturers and different formulas used in laboratories. In this study, different IC_50_ values were detected compared to other similar studies [22]. Due to the inevitable side effects of chemotherapy agents, there has been an increase in the tendency toward alternative products. New combinations are being tried especially to protect healthy cells and provide tumor shrinkage. In this study, GA, which has been revealed to have anticancer properties in many types of cancer, was preferred as an alternative product that would reduce the side effects of an effective and highly cytotoxic agent such as DOX in the treatment of cervical cancer. With their combination, the dose of chemotherapy agent used could be lower and effective anticancer properties would be revealed with GA, which has antioxidant properties. Indeed, our findings supported this. DOX and GA duo could be good alternative anticancer agents in uterine cancer.

Ou et al. explained the ability of GA to induce G2/M arrest by changes in cell cycle regulators in the human bladder transitional carcinoma cell line [23]. This effect is controlled by a family of cyclin/cyclin-dependent kinase (CDK) complexes and CDK inhibitors (CDKIs) responsible for cell cycle regulation [24]. The G2/M transition is mainly regulated by the sequential activation and deactivation of CDK regulatory proteins and cyclin complexes. Cdc2 is also known as Cdk1 and initially forms a complex with Cyclin B1 to move the cell from the G2 to the M phase [25]. The main regulators of the intrinsic pathway are the Bcl-2 family pro and anti-death members [26]. These proteins are located in the mitochondria and control the release of P53, an important tumor suppressor that can modulate key checkpoints in both extrinsic and intrinsic pathways [27].

In our study, GA and DOX were observed to induce apoptosis according to both MTT analysis and NucBlue staining method results. Also, to understand the role of GA and DOX in inducing apoptosis, P53 and Bax expression were detected by RT-qPCR assay. The GA/DOX combination showed the highest level of P53. At the same time, Bax expression, another proapototic gene, was observed at the highest level in the DOX and DOX+GA groups. P53 is required to maintain G2 arrest following DNA damage because tumor cells lacking this protein undergo mitosis with accelerated kinetics [28,29]. Therefore, the induction of P53 is important for the suppression of tumor proliferation. The ability of GA to upregulate P53, seen in our study, has been detected by other researchers on different cancer cells. It has been reported that GA exhibits anticancer properties by increasing P53 gene expression via the mitochondrial apoptotic pathway on human SCLC H446 cells [27,28,29,30]. The potentiating effect of the DOX+GA combination seen in our study was reported by Sánchez-Carranza et al. It has also been reported that it is effective on human ovarian carcinoma A2780 cells (drug-sensitive) and A2780AD cells (multidrug-resistant ovarian cancer) in combination with GA. Sánchez-Carranza et al. explained this by the ability of GA to potentiate Paclitaxel-induced G2/M phase arrest. Researchers argued that it may be due to the excessive production of ROS by GA, which triggers proliferation inhibition and plays an active role in stopping the G2/M phase, and the chemotherapy agent Paclitaxel triggers extracellular signals [31,32,33].

The tumor suppressor proteins P53 and Bax are responsible for regulating gene expression and activating DNA repair mechanisms, cell cycle checkpoints, and the apoptosis response. DNA-damaging agents induce P53, which plays a leading role in directly activating the pro-apoptotic bax gene to activate the apoptotic program [34,35,36,37]. In this study, it was determined that the GA/DOX combination increased the expression of P53 and Bax in HeLa cells.

Despite the promising findings of this study, several limitations should be acknowledged. First, the experiments were conducted exclusively on HeLa cell lines, which may not fully represent the complexity of cervical cancer in vivo. Additionally, the study focused primarily on the P53/Bax signaling pathway, leaving other potential apoptotic pathways influenced by GA unexplored. Further in vivo studies and investigations into other signaling pathways are necessary to fully elucidate the therapeutic potential and broader mechanisms of GA in cervical cancer.

## 5. Conclusions

Our study provides compelling evidence that the GA/DOX combination exerts apoptotic effects in HeLa cell lines through modulation of the P53/Bax signaling pathway. By elucidating the molecular mechanisms underlying its apoptotic properties, we have contributed to a deeper understanding of the therapeutic potential of GA in cancer treatment. These findings highlight the significance of natural compounds in combating cancer progression and underscore the importance of further research to explore the clinical applications of GA and similar phytochemicals. Ultimately, our study contributes to the growing body of knowledge aimed at developing more effective and targeted therapies for cancer management.

## Figures and Tables

**Table 1:** The primers used to investigate changes in the expression of genes are in the order of 5′–3′.

P53: F: CACGAGCGCTGCTCAGATAGC, R: ACAGGCACAAACACGCACAAA
BAX: F: TTCATCCAGGATCGAGCAGA, R: GCAAAGTAGAAGGCAACG
β-Actin: F: CCTCTGAACCCTAAGGCCAAC, R: TGCCACAGGATTCCATACCC
GAPDH; F: CGGAGTCAACGGATTTGGTCGTAT, R: GCCTTCTCCATGGTGGTGAAGAC

## Data Availability

All data that support the findings from this study are available from the corresponding author upon reasonable request.
